# A Case of Infective Endocarditis in a Middle-Aged Patient With Trisomy 21 and an Incidentally Discovered Patent Foramen Ovale (PFO)

**DOI:** 10.7759/cureus.61106

**Published:** 2024-05-26

**Authors:** Sinead N Bhagwandeen, Girma M Ayele, Amoafo D Boampong, Victoria J Bhagwandeen, Niyati Grewal, Christopher C Williams, Renee Skific, Benedicta Arhinful, Harminder Sandhu

**Affiliations:** 1 Internal Medicine, Howard University Hospital, Washington, USA; 2 Biology, College of Natural and Health Sciences, University of Tampa, Tampa, USA

**Keywords:** congenital heart disease, patent foramen ovale, down's syndrome, trisomy 21, infective endocarditis

## Abstract

Trisomy 21 often leads to cardiac complications, usually associated with congenital heart disease, such as atrial septal defects, ventricular septal defects, and patent ductus arteriosus. This case describes an unexpected instance of infective endocarditis (IE) in a middle-aged patient with an incidentally discovered patent foramen ovale (PFO). The common risk factors for IE include previous valve surgery, artificial heart valves, pacemakers, prior IE, congenital defects like bicuspid aortic valve, IV drug use, and the congenital defects mentioned earlier.

## Introduction

Down Syndrome (DS) or trisomy 21 is associated with a 40 to 50 times greater likelihood of congenital heart disease (CHD) than in the general population [1]. The most common cardiac complications of trisomy 21 usually are a part of the sequelae of underlying CHD, including atrial septal defect, ventricular septal defects (VSDs), secundum atrial septal defects, patent ductus arteriosus and, less commonly tetralogy of Fallot (ToF) [2]. This case highlights an unsuspected case of infective endocarditis (IE) in a middle-aged patient with an incidentally discovered patent foramen ovale (PFO). The usual risk factors for IE include previous valve surgery, artificial heart valves or cardiac devices such as pacemakers, prior IE, congenital bicuspid aortic valve, IV drug use, and congenital defects, as aforementioned.

## Case presentation

This is the case of a 55-year-old male with a prior history of Down’s syndrome (trisomy 21) and type 2 diabetes mellitus (DM) who presented with altered mental status.

**Figure 1:**
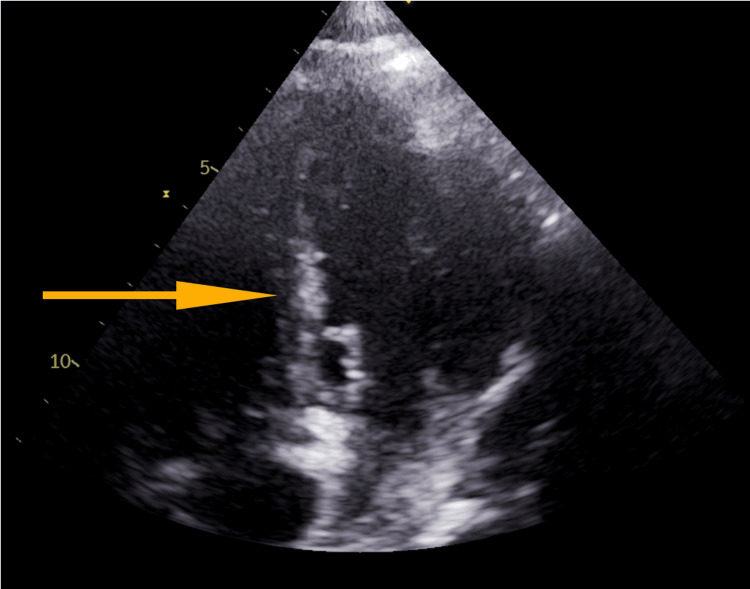
Suspected mitral mass during trans-thoracic ECHO (TTE)

He presented with increased drowsiness which was apparent from his baseline. The patient was non-verbal at baseline and could not perform his activities of daily living. On physical examination, the patient was febrile with a temperature of 101.4F and the rest of the examination was non-revealing. Broad-spectrum antibiotics, which consisted of Vancomycin and Zosyn, were initiated after a septic evaluation was obtained. After 24 hours, two (2) pairs of blood cultures revealed gram-positive cocci in clusters, reported as methicillin-resistant *Staphylococcus aureus* (MRSA). Further workup included a trans-thoracic ECHO (TTE), which demonstrated normal left ventricular (LV) size and function and an echo density that was described as "not well visualized," but was suspicious for a mitral valve mass or echo density (Figure 1). Separate blood cultures one day post-initiation of antibiotics remained positive, despite continuing Vancomycin.

**Figure 2:**
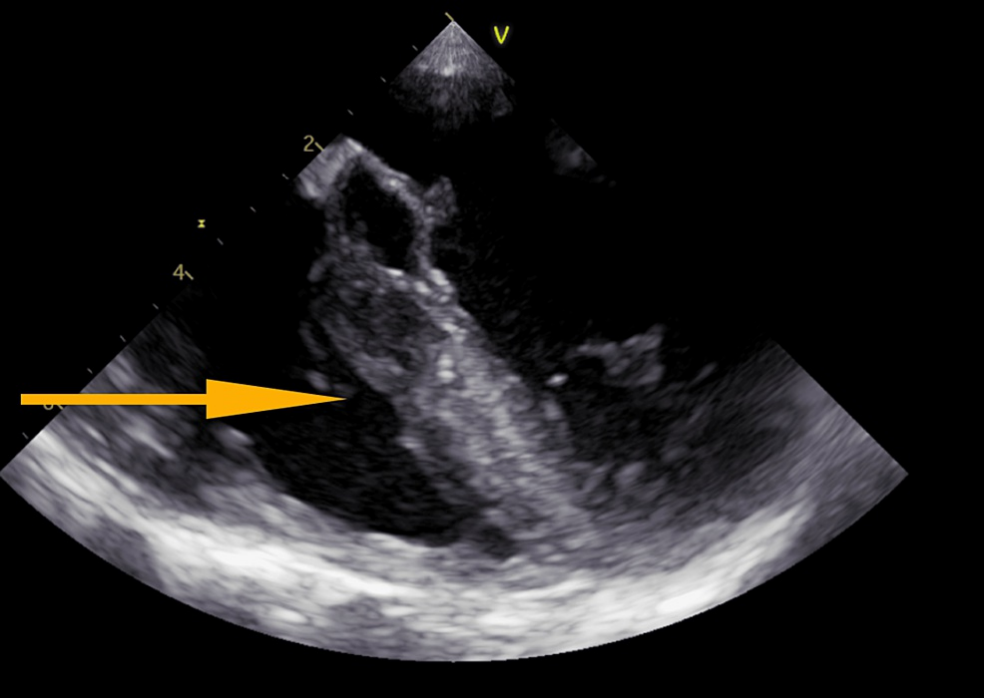
Echo density/mass seen on the mitral valve during trans-esophageal ECHO (TEE)

**Figure 3:**
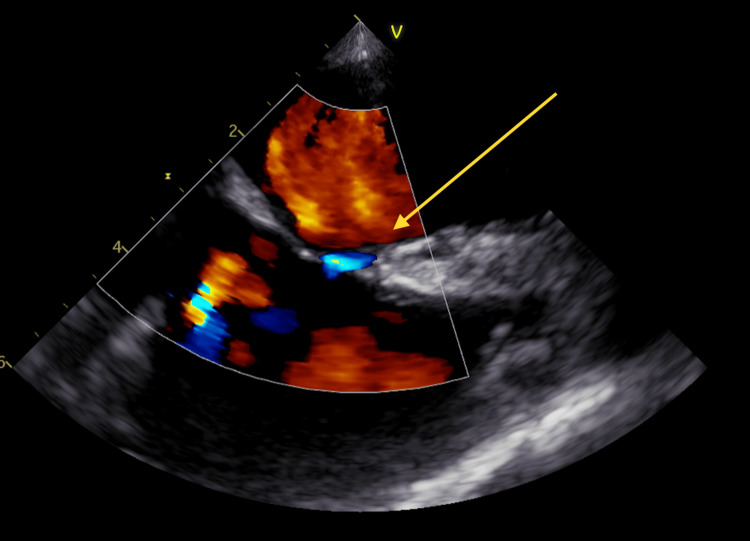
Patent foramen ovale (PFO) demonstrated using color flow Doppler during TEE TEE: trans-thoracic ECHO

Trans-esophageal ECHO (TEE) was subsequently done (Figure 2), the report of which showed “A small echo-density noted on anterior mitral valve leaflet." Differentials included vegetation versus focal thickening of the leaflet. The echo density did not demonstrate independent motion as would typically be expected of vegetation; however, given the patient’s clinical picture of bacteremia, endocarditis should have been considered. Mild mitral regurgitation was also observed. There was a PFO seen by color Doppler (Figure 3); a bubble study was attempted but was not performed due to technical difficulties with IV administration. The aortic valve was reported as a tri-leaflet and functioned normally.

Infectious disease specialty was consulted and the patient was initiated on antibiotic therapy with Daptomycin based on blood culture sensitivities. The patient’s clinical status improved within two days after Daptomycin initiation. He was no longer febrile and blood cultures were repeated two days later which no longer demonstrated bacteremia.

## Discussion

DS substantially increases the risk of CHD compared to the general population, with a 40 to 50 times higher likelihood. Approximately half of infants born with DS are diagnosed with CHD, whereas approximately 1% of the general population is affected. However, the precise incidence of CHD in individuals with DS varies widely from 23% to 79% across different geographical locations, even in studies designed to minimize referral bias. Environmental factors like maternal smoking, obesity, and inadequate folic acid supplementation during pregnancy are known to contribute to this increased risk, making the incidence of CHD in DS within a population-based study dependent on the maternal characteristics of the study cohort. Interestingly, despite these variations, the incidence of CHD in DS appears to have remained relatively stable over time [3].

Among the various CHD types observed in DS, atrioventricular septal defect (AVSD) is the most common, followed by isolated ToF in approximately 13% of cases, a combination of AVSD and ToF in about 9% of cases, and isolated VSD in 4% to 17% of the cases. Transesophageal echocardiography is recommended for more precise visualization of vegetation, especially in complex types of CHD in adults with CHD (ACHD patients). Recent trends noted by Bergström and colleagues indicate a shift toward simpler CHD lesions in individuals with DS. This shift may be attributed to better survival rates for simpler defects or a higher rate of prenatal diagnosis, leading to a greater likelihood of pregnancy termination when more complex defects are identified [3,4]. Increased survival of children with CHD due to the use of conduits and prostheses in corrective surgery may have also contributed to an increasing incidence of IE [3,4].

It's important to note that the threat of IE remains significant among individuals with CHD, whether their condition is unrepaired, palliated, or corrected. The incidence of endocarditis in adults with CHD has been documented at 11 per 100,000 person-years, marking a notable rise compared to the general population, which typically reports rates ranging from 1.5 to 6.0 per 100,000 patient-years [5,6]. CHD lesions and recent cardiac surgery within the preceding six months exhibit the strongest correlation with the onset of IE. Patients with left-sided lesions and endocardial cushion defects are at increased risk of IE, even among children without a history of valve surgery or prior IE, both of which are presently classified as high-risk factors for IE [5,6].

Notably, CHD-associated IE mortality has decreased substantially to 10% because of improvements in the diagnosis of IE, antimicrobial treatment, cardiac surgery, and interventional therapy. Antimicrobial therapy and surgical management of IE remain challenging, but the outcome of CHD-associated IE from the neonate to the adult is better than in other forms of IE [6,7].

## Conclusions

This case serves to demonstrate the utmost importance of evaluation for congenital heart defects when assessing any patient with​​ trisomy 21 or DS. This patient had never received a diagnosis of CHD prior to this presentation of sepsis and altered mental status, where he was diagnosed with IE. It also serves to emphasize the use of TEE in assessing these patients, particularly when the TTE is negative or even inconclusive, as was found in this case. This case also serves as a reminder that there is a greater prevalence today in adults with congenital heart defects, which may be attributed to better survival rates for simpler defects or a higher prenatal diagnostic rate.
